# Stand up and Fight: A Case Study of a Professional Rugby Club Negotiating a COVID-19 Crisis, a Talent Development Perspective

**DOI:** 10.3390/sports10080124

**Published:** 2022-08-16

**Authors:** Ian Costello, Sarahjane Belton, Áine MacNamara

**Affiliations:** 1 Munster Rugby, V94 X9VK Limerick, Ireland; 2 School of Health and Human Performance, Dublin City University, D09 W6Y4 Dublin, Ireland

**Keywords:** challenge, development, coherence, talent development

## Abstract

A wealth of literature examines the role of challenge from an individual psychological perspective, but research investigating how a talent development system can proactively support athletes to successfully meet the ever-increasing demands of top-level professional sport is less prevalent. This study takes advantage of a naturally occurring but highly atypical developmental challenge as a result of COVID-19 to examine factors influencing the efficacy and effectiveness of the talent development pathway at Munster Rugby. Players and staff (*n* = 12) took part in semi-structured interviews exploring their experiences of the build-up to the event, the game itself, and the impact post-event. The data were subsequently analysed using Reflexive Thematic Analysis. Players and coaches highlight the groundwork undertaken to establish alignment and coherence, both horizontally and vertically across the talent development environment, and how this contributed to navigating the challenge successfully. The findings support the necessity of both the player and the talent development system being prepared to enable players to perform at the highest level. The findings point to an overlap between the development and performance phases of a player’s journey and the need to integrate short- and long-term objectives within a talent development system.

## 1. Introduction

Over the last 30 years, considerable attention has been paid to the broad area of talent development in sport, with research focused on the principles underpinning effective talent development environments [1] incorporating a holistic environmental approach [2], factors impacting the development of high potential young players [3], the talent development system [4], talent development coaching [5], and talent development coaches [6]. Much of the research has been positioned against a pragmatic framework [7], motivated by the desire to generate effective answers to talent development problems directly applicable in the ‘real world’. Collins and Kamin [8] refer to this as science *for* sport as opposed to science *of* or *through* sport; an approach underpinned by a motivation to generate practically meaningful knowledge.

Reflecting the need for coherence as an integral part of the talent development pathway [9], there is increasing consideration of how best to design and operationalise the talent development system. Although there is a well-established positive correlation between financial investment and performance outcomes in sport [10], some successful sports systems benefit from significant financial investment (e.g., in excess of GBP 100 million investment by UK Sport, 2015), while others still succeed with significantly less resources. In the latter case, appropriate talent development pathways are critical to this success, and must be carefully resourced and structured to optimise the efficacy and output of their pathway. In professional sports such as rugby, the talent development pathway is frequently structured as an academy that is structurally and systematically linked to the club’s performance pathway. The objective of an academy in these settings is primarily the development and nurturing of high-potential players to facilitate their progression to first-team performance status [1]. To achieve this outcome, it is important that there is a coherence between what is happening at different stages of the talent development pathway [5], along with a shared understanding of processes and behaviours [11], at different phases to facilitate this movement.

### Coherence in Talent Development Systems

It is widely accepted that talent development is a complex, dynamic, and non-linear process [9], with effective talent development environments characterised as offering individualised development, deploying long-term aims and methods, and focusing on a breadth of psycho-behavioural and psycho-social skills with coherence across stakeholders [12]. As such, effective talent development systems are characterised by inputs that are complementary, framed, and structured against long-term agendas. A strategic strength of an effective talent development system is a cohesive philosophy between stakeholders operating both horizontally (i.e., within a particular stage of development; e.g., a professional sports academy) and vertically (i.e., between stages of development; e.g., across the development system) [13], where shared values, expectations, and behaviours support the development of high-potential players [14]. In reality, however, there is often a diverse range of philosophies, beliefs, and methodologies being applied to many aspects of talent development, with what is best described as a lack of joined-up thinking. A lack of horizontal and vertical coherence, along with a lack of shared mental models [15], has been identified as a barrier to optimising the TD experience [5], especially where relationships are suboptimal between stakeholders, [16].

In this regard, a lack of organisational proximity and communication within a talent development system has been suggested to hinder player development [9]. Navigating coherence becomes even more complicated when players co-inhabit multiple pathways (e.g., club, academy, senior team), unless there is clarity in the message and support received at each level. Of course, coaches and players operating within a performance setting and a development environment will, by necessity, have different aims and objectives; the former focused primarily on outcome success, and the latter on longer-term development. Although the talent development system is conceptualised as a pathway, in reality, academy, club, and performance coaching and development often happen independently [17], with coaches and players operating in a siloed manner without shared goals and visions [4]. This is further complicated by the fact that high-potential young players rarely reside exclusively in either the performance or development worlds, but instead regularly move between these environments. Reflecting this, there have been calls to consider how the talent development system is designed to optimise the methods, structures, and opportunities afforded to high-potential athletes to allow them to navigate in-career transitions [1].

The coherence and effectiveness of the talent development system may be especially tested when young players are presented with non-normative transitions that stretch their skills and capabilities. In fact, the role of challenge in talent development has drawn considerable attention (and challenge!) since seminal work in 2012, which identified that, ‘talent needs trauma’ [18]. Traditional talent development approaches were often based on optimising the support offered to high-potential young athletes and minimising challenges for an athlete in order to smooth their development pathway. However, despite the face validity of such an approach, there is a growing research base suggesting that experiencing challenges on the development pathway, and then debriefing and learning from that experience, supports the development of talent, and therefore, young athletes benefit from a variety of challenges/bumps that they encounter on the ‘rocky road’. Although some transitions are well flagged e.g., normative transition from youth to senior teams, transition into and out of academy settings [19]; high-potential young players often encounter a range of non-normative and unexpected transitions as part of their development journey. Wylleman & Lavelle [20] proposed a developmental model on transitions faced by athletes that takes a linear perspective from beginning to end, reflecting the nature of normative transitions at athletic, psychosocial, academic, and vocational levels. Stambulova’s [21] Athletic Career Transition model focuses on the importance of an effective coping response for an athlete to make a successful transition from one developmental stage to another. These transitions can be challenging if the athlete does not have a well-developed skillset (physical, technical, tactical, and psycho-behavioural) and support system. In this respect, the importance of psycho-behavioural skills as the mechanism that supports the navigation of the challenge, and as the foundation for further learning and development as a result of the challenge, has been well explored [22,23]. Critically, the importance of identifying developmental challenges that can be optimally deployed to develop this skillset and maximise growth is recognised [5]. Although some attention has focused on the purposeful operationalisation of artificial ‘speedbumps’ on the pathway [23] as a means of ensuring the skills are tested, taught, practised and embedded, it is also apparent that high-potential young players will encounter a range of naturally occurring challenges as part of their trajectory. Athletes’ and coaches’ experiences of navigating and learning from such naturally occurring challenges represent a naturalistic lab to test the efficacy of the talent development system, and the ability of young athletes to navigate and grow from challenges. Once such, a naturalistic lab opportunity arose when Munster Rugby lost 48 players and staff from their senior team for a European Cup match in 2021, with young academy and National Talent Squad (NTS) players from the Munster pathway required to ‘step into the breech’. The purpose of this current study, therefore, was to provide an in-depth and rich understanding of key stakeholders’ experiences of navigating this naturally occurring challenge on the talent development pathway.

## 2. Materials and Methods

In this paper, we present a case study of a talent development system within a professional rugby club (Munster Rugby), with a particular focus on how one particular challenge was navigated; namely, the loss of 48 players and staff from the senior team for a European Cup match in 2021. The case study approach allowed us to explore this phenomenon in context, using a variety of data sources [24]. An ethnographic approach enabled us to capture the phenomenon from the viewpoint of the coaches and players involved in the match by using fieldwork, observation, and informal interviews to gain a rich understanding of the experiences within Munster Rugby and examine the complexities of the interactions and experiences in the group. The prolonged and in-depth engagement required by an ethnographic approach was facilitated by the first author, who was employed as the academy manager at the club throughout this period.

### 2.1. Context

Munster Rugby was founded in 1879 and is one of four provincial professional rugby teams in Ireland. Munster has a proud history in the European cup, reaching or surpassing the semi-finals 14 times since the tournament’s inception in 1995. The most successful era in the club’s history resulted in the club lifting the trophy on two occasions, in 2006 and 2008. It has a strong tradition of competing against international touring sides, with the most famous victory being against the touring All Blacks in 1978. In December 2021, during the worldwide COVID-19 pandemic, Munster Rugby travelled to South Africa on a two-game tour as part of the United Rugby Championship. Immediately on their return, they were scheduled to play Wasps in the first round of the Heineken European Champions Cup. Following Covid -19 testing, 14 players and staff were forced to remain in quarantine in South Africa, while a further 34 members of the squad were required to be isolated in Ireland for ten days upon their return. This resulted in all members of the squad who travelled to South Africa, both players and staff, being unavailable for preparation (and selection) for the game against Wasps. A small number of senior contracted players (*n* = 10) had not travelled with the squad to South Africa (seven staying due to participation in an Irish Rugby series of matches, while three were returning from injury). To fulfil the Wasps fixture, Munster were forced to select a squad comprising of those ten senior players, along with young academy and NTS players chosen from the Munster pathway. Fourteen players were selected to make their European debut in this game, with twelve of those representing Munster at senior level for the first time. In addition, the senior coaching staff were unavailable due to COVID-19 protocols meaning that the academy staff led preparations for the game, and coached and managed the team on game day itself.

### 2.2. An Insider Perspective

In July 2021, six months prior to the game in question, I (first author) had been appointed as Munster Academy Manager, after returning from coaching professionally in the UK for five seasons. I had previously held positions in Munster Rugby including as a senior team assistant coach from 2010–2016 and as academy coach from 2008–2010. Prior to this, I had coached at each stage of the Munster pathway from youths and schools level through to All-Ireland League club level. With all Munster senior coaches forced to isolate as described above, I became the defacto head coach for the Wasps game in December 2021. Coincidentally, my last three years coaching in the UK, immediately prior to my return to Munster, were spent working as an Assistant coach with Wasps Rugby, at which time I had also enrolled on a Professional Doctorate exploring talent development practices in rugby. Without doubt this added extra spice to what was already an intriguing mixture.

The challenges of this unprecedented situation provided a test of Munster Rugby’s talent development pathway, and an opportunity to explore key stakeholders’ experiences and perceptions of a unique developmental challenge. It also provided a real-life case-study to examine the efficacy of the principles underpinning the Munster pathway; principles that the academy staff had spent the previous six months aiming to instil and embed across Munster’s talent environment. These principles are outlined in the following section.

### 2.3. Principles Underpinning the Munster Pathway

Starting in my role as Munster Rugby Academy Manager, I prioritised a number of principles that I felt were essential to developing an effective TDE at Munster Rugby. These principles were based on a combination of my experiences in my coaching career to date, intimate knowledge of the Munster Rugby landscape, and knowledge acquired from TD research I had carried out up to that point on my Professional Doctorate journey. The build-up to, and the Munster versus Wasps match itself, also provided a unique opportunity for a ‘temperature check’ on the efficacy and impact of these principles in terms of preparing staff and players to embrace (and successfully navigate) many of the challenges encountered during this period. These principles were:

- Understanding and aligning the multiple stakeholders [4,25];
- Vertical and horizontal coherence, and shared mental models [5];
- Supportive but challenging environment, with players moving quickly through different stages of the pathway [18,26];
- Emphasis on developing skills, players, and staff [12,14].

#### Munster, a Complex Landscape

Munster Rugby is a particularly complex landscape with many challenges to creating an effective, coherent TD pathway. Taylor & Collins [5] described the high-pressure milieu of professional sport as being a significant barrier to coherence, with cultural and socio-political realities often having a negative impact on the long-term success of a Talent Development Environment (TDE). In Munster there are long standing historical and political considerations that impact decision making at all levels of the organisation. The province consists of a vast geographical region and incorporates multiple stakeholders across the entire TDE.

Vertically, the academy is completely aligned with the senior team, follows the senior programme, and operates out of the same training base in the Munster High Performance Centre under the remit of the Academy Manager. Below this level, the National Talent Squad (NTS) comprises of a group of players that have been identified as potential academy entrants, with a different training base, a separate training schedule, and an entirely different group of coaches (partly due to Covid -19 ‘bubbles’). The management of this programme also falls under the remit of the Academy Manager.

Horizontally, the clubs and schools are key stakeholders in the TDE and are the primary feeders of the Munster pathway. The majority of academy and NTS players play club rugby in the amateur All-Ireland League each week. The European Champions Cup represents the highest standard of club rugby in Europe, and it is incredibly rare that a player who is not a contracted senior team player would be selected to play in the competition. On the occasion examined in this paper, 13 non-senior players (i.e., players from the Munster pathway) were selected in the match day 23 for the European Champions Cup match against Wasps, presenting a challenge of unprecedented magnitude.

### 2.4. Data Sources

Data for this study was collected by the first author using a combination of field notes and informal interviews. Ethical approval for the study was granted by the authors’ institutional Research Ethics Committee DCUREC/2022/015.

#### 2.4.1. Field Notes

The first author recorded daily observations and reflections of coaching and strategic decisions within his environment from July 2021 to February 2022. These consisted of oral and written records of incidents, events, documents, unusual occurrences, meetings, decisions, and observations. Reflecting the importance of prolonged engagement in ethnographic research, this study was completed over thirty-two weeks of daily contact.

#### 2.4.2. Informal Interviews

The first author carried out a series of internal group and individual interviews with players (*n* = 11) and coaching staff (*n* = 4) involved in the Wasps-Munster match preparation and game day. These interviews were conducted informally at the Munster Rugby training base at times that were convenient for the participants. All participants were involved in the preparation for the Wasps-Munster match either as NTS players, academy players, first team players or coaching staff. The interviews lasted between forty-five and sixty minutes, with the first author also following up with individuals post-hoc for clarifications and further probing as part of a member reflection process [27]. During the interview, participants were asked questions about the preparation for the game, the environment during the two weeks’ preparation, their experience of the game, how groundwork in the previous six months supported them during the event, and if they had experienced any post-event impact.

### 2.5. Data Analysis

Firstly, it must be acknowledged that data analysis in ethnographic research is complicated, and therefore no single method is universally accepted as a strategy for data analysis [28]. As such, an iterative process of theme building was undertaken [29] using the dataset as a whole. As a first step, text from field notes, observations and interview transcripts were labelled and then categorised and sorted according to a theme using a constant comparative method of analysis [30,31]. This step involved multiple reviews of the data and then coding for similarities, differences, groupings, patterns, and items of particular significance [32].

### 2.6. Trustworthiness and Integrity

This study was underpinned by a pragmatic approach to research, and therefore decisions about methodology and data analysis were chosen with these philosophical underpinnings in mind. Of course, given the nature of this study, it was important to consider and implement steps to ensure the trustworthiness and integrity of the data collection and data analysis. Reflecting Bradshaw et al.’s [33] suggestion, a number of quality markers were implemented:

1. Firstly, reflecting the importance of in-depth and prolonged engagement and to ensure the credibility of the findings, data were collected over 32 weeks by the first author, who held a thorough and rich understanding of the context;
2. Triangulation of data was employed using a range of data sources, including observations, interviews, focus groups, and reflections, in order to increase the rigour of the data;
3. Throughout the study, from conception through to write-up, critical friends were employed to address the trustworthiness and credibility of the findings;
4. Rich and in-depth accounts of each theme are offered using contextualised data to offer the reader an opportunity to consider the transferability of the findings to other contexts.

As described earlier, the first author was appointed academy manager at the start of the data collection process, thus bringing an insider perspective, deeper knowledge, and interactions to the research [34]. Of course, this also presented some potential challenges to the rigour of the research. To counter this, reflectivity was employed to enhance rigour [35], with the first author keeping a reflective diary to record their thoughts, behaviours, and actions during the data collection. This process supported the development of self-awareness, criticality, and flexible thinking during the data collection and data analysis phases of the research.

## 3. Results and Discussion

The results derived from the multiple sources in this ethnographic study provide an in-depth and rich understanding of key stakeholders’ experiences of navigating a naturally occurring challenge on the talent development pathway at Munster Rugby. The three key themes, as highlighted in Table 1, were:

- Groundwork to establish alignment and coherence on the pathway;
- Preparation for the Munster-Wasps game;
- Impact post challenge.

Each theme is supported using vignettes to capture the participants’ voices in portraying events.

### 3.1. Groundwork to Establish Alignment and Coherence on the Pathway

As outlined previously, prior to the Munster-Wasps game, a number of key principles were prioritised, many of which were based around establishing alignment and coherence on the pathway. The groundwork undertaken in the six months prior to the Munster vs. Wasps games certainly appears to have been an influential factor in how players and coaches successfully navigated the challenge. The complete integration and alignment of the senior and academy training programmes meant that academy players trained on a full-time basis with the senior team, and in addition, the academy coaches had full access to all senior meetings and sessions. An academy player who played in the match talked about how seamless the transition was as a result:

“*We train the exact same as the seniors. If we hadn’t been training week in week out with the seniors we wouldn’t have been aligned, we wouldn’t have known all the roles and every single call, all as one group the whole time, it was seamless really*”.

This vertical alignment within the club [9] enabled the academy players and coaches to get on the same page quickly when preparing for the game. The groundwork that had been carried out in relation to establishing shared mental models [5] (e.g., game plan, playing details, coaching practices) proved to be invaluable when presented with so many potential challenges during the build-up to the game.

Recent improvements to connections and communications that had been made between senior and academy coaches were considered a positive factor in alignment between the groups [13], and proved important when it came to making informed selection decisions in assembling a squad that included so many players outside the traditional senior squad. An academy coach highlighted the following:

“*We started the practice of having regular pathway meetings with the senior coaches (every 6 weeks) so that they were familiar with the players coming through but also so we have a shared understanding of what we were looking for in players*”.

Another academy player referenced the fact that, unlike in the past, academy players were now included in senior social functions, which “*really helped us all to work together*” in challenging circumstances during the build-up to the game when a group of players who had never played together needed to come together as a team, connect and build relationships quickly.

Connecting the pathway was a critical part of the groundwork in establishing alignment and coherence from top to bottom [36]. The academy coaches consistently work at all stages up and down the pathway, and had spent eight weeks the previous summer coaching the NTS players. This particular group of players were pivotal during the two-week preparation leading into the game, and in fact, two NTS players (sub-academy) were named in the match-day squad. This regular contact between academy coaches and pathway players enabled coaches to develop strong relationships with these players and exposed these pathway players to the training methods and practises employed at academy and senior level. An academy coach talked about the value of this:

“*The sessions we did in the summer (with the NTS players) was very similar to what they experienced during the two weeks. They had trained that type of format and they were familiar with the academy coaching staff. Coaching up and down the pathway stood to us in the prep*”.

The deliberate practice of academy coaches and pathway coaches regularly working together meant that, in relation to both coaching philosophies and coaching practices, there was a shared mental model and a common coaching framework [6], which allowed everyone to get on the same page quickly.

“*Academy coaches and the NTS and Talent coaches worked closely together for the 6 months leading into game, so when all the players and staff came together to prepare for the Wasps game we were on the same page*”.

Striving to create a high-performing environment in the academy, based on high challenge and support, was recognised as an important piece of the groundwork undertaken in the previous six months that enabled the coaches to operate effectively and cohesively under unusually demanding circumstances in the build-up to the Wasps game [37]:

“*Our coaching environment (academy) is very open and honest and based on challenge and support, we have each other’s backs but we challenge each other to be operating at the highest standards*”.

A process facilitated by the academy’s performance psychologist, began six months prior to establish a clear purpose for the academy with an agreed set of values to guide behaviours. At the core of the purpose was a focus on raising standards across the pathway and a commitment to long-term development versus a short-term performance focus.

“*As a newly formed academy team (Multi-disciplinary Team, MDT) we had a focus on raising the standards right across the board in the whole Pathway. This began with establishing a clear purpose and an agreed set of values and this was brought to life through our behaviours. We aligned to a key message of the whole Munster organisation ‘we rise by lifting each other’*”.

As described earlier, Munster is a complex landscape, and the local schools and All-Ireland League (AIL) clubs are key stakeholders in the TDE. Horizontal alignment between Munster and these important stakeholders was a specific focus upon coming into the Academy Manager role six months earlier, with the aim of establishing strong relationships and building on them throughout the season [38]. The majority of players that are in the senior squad have come through the academy and from local clubs and schools. Most academy players will play their rugby each week in the AIL league, and the importance of aligning that experience to the academy was stressed by one of the coaches:

“*We communicated with each AIL Head coach twice per week regarding Academy players and we offered the coaching support to all the clubs. This was vital when we needed club players to prepare for the Wasps game, we had incredible support with releasing players to train etc.*”.

One academy coach spoke about the process of mapping out more regular visits to AIL matches to scout academy and pathway players and to build relationships with club coaches; “*Each month we mapped out a schedule so that coaches rotated regularly to watch different club games*”. This process proved invaluable when it came to making informed selections for the Wasps game and also getting cooperation from club coaches to release their players to train for the game, with another academy coach noting: “*Increased visibility of all the academy coaches at club games helped to get to know coaches and players which helped with selection for Wasps*”.

### 3.2. Preparation for the Wasps Versus Munster Game

As the COVID-19 situation unfolded and it became apparent that 45 senior players and staff would be unavailable for the Munster-Wasps game, a number of alignment meetings with coaching staff and players were organised to navigate the unique challenges that were presented. The coaching staff made a number of decisions to employ specific strategies based on bringing an unfamiliar squad together, building connections and integrating the group to get them on the same page as quickly as possible, to optimise the two-week preparation for and performance in the Wasps game.

One of the first decisions made by the coaching staff was to organise a meeting with the senior players that had not travelled to South Africa, with the purpose of outlining a ‘dual management’ model (shared ownership) between the coaches and senior players on all aspects of game preparation [39]. This involved empowering senior players and giving them the responsibility to lead the group and provide input across both the on-pitch and off-pitch components of preparation. An academy coach recalled that “*Seniors players had input into game plan design and session content and they presented throughout the two weeks in training reviews and opposition previews*”. The senior players bought in to this approach and invested heavily in preparing the squad for the game. An academy player observed the impact this had on the wider group:

“*The coaches gave the senior players a licence to help take the sessions as well, there was an emphasis that they would lead from the front. It gave us confidence that they trusted us and the coaches trusted us. There was real sense of trust around and that brought a closeness*”.

This approach is based on an approach employed by the All Blacks (New Zealand rugby team) as documented by Hodge et al. [39], and is consistent with the coaching philosophy of the academy coaches at Munster. Many of the principles associated with dual-management are consistent with autonomy-supportive coaching [40], and emotional leadership coaching [41]. One of the senior players who was part of this leadership group captured the impact that it made during the preparation:

“*If you empower players it creates a different type of trust and bond in the group, coaching each other, not you (coaches) deciding what we are doing but coming up with it together*”.

Following on from the theme of player empowerment, the participants noted the impact of a buddy system [42], between each senior player and a younger academy or pathway player that was implemented during the preparation phase. This provided a mechanism for connecting players that were either new to the group, or had never played at European Cup level before, with senior and established players. An academy coach noted that:

“*Every senior player was partnered with a young player, took him under their wing and supported him throughout the two weeks right up to presenting the jersey to their ‘mentee’ the night before the game*”.

The influence of role models is well documented [43,44], with proximity and access to role models documented as one of the key Environmental Success Factors (ESF) in talent development environments.

The buddy system concept was deployed to optimise the resources available to help all the new players get up to speed with Munster game principles and specific details in the game plan. This was particularly important as key coaching staff were also absent from the preparation phase. This dual-management approach complemented the role of the senior players by giving them more responsibility in preparation for the Wasps game while also serving to build connections between players that had never played together. In addition to the technical and tactical benefits in relation to match preparation, an academy player talked about how he was inspired by working so closely with his role models [12]:

“*It was kind of inspiring as well because you’re having intimate conversations about your game with players who you grew up watching that you really respect and admire*”.

It is worth noting that, in addition to the short-term benefits observed from this mentoring approach, the relationships forged between the players remained long after the Wasps game; this is described in a later section as part of the third higher order theme.

From the outset, there was a general acceptance from all participants that there were going to be aspects of preparation that would be far from perfect (in fact, it became a bit of an inside joke), and to this end, both players and coaches accepted that they were in a highly untypical situation and, as described by one coach, embraced a theme of ‘*improvise, adapt, overcome*’ for the two weeks prior to and during the match. For example, the coaches observed the mentoring role the senior players played in relation to creating an environment that accepted that mistakes would be made in these circumstances; “*the senior lads were great at helping the younger players move past mistakes, it happens, learn, move on*”. This approach to the game appeared to be supported by the steps taken to integrate the squad and build trust and connections as quickly as possible, which was a primary focus of initial preparation. An academy coach pointed out the importance of this; “*Every meeting and session always included an element of connecting players, building relationships, we had to bring the squad together, get on the same page quickly*”. The team psychologist played a major role in connecting the squad early in the preparation phase. For both the inexperienced players and senior internationals alike, this match represented one of the biggest challenges of their rugby careers to date, but steps were taken to ensure it was presented as an opportunity to write their own history rather than as an insurmountable challenge. As one example, the team psychologist organised formal team meetings centred on developing team culture to augment the technical and tactical preparation. Developing this vision through formal meetings and facilitation by team staff was identified as a critical moment in the team’s preparation, as highlighted by a senior player; “*The psychologist’s meeting had a big role to play in that, the connection and vulnerability piece settled everyone as a group. We left that meeting feeling like a real team*”.

Central to preparation for the Wasps game was creating a positive, task-focused environment [45]. Due to the unusual circumstances surrounding the game, there was plenty of distraction, media, etc., that had the potential to affect the preparation of the young squad. There was ongoing discussion within the group to stay in the present, concentrate on the current task at hand, and continuously reinforce the importance of ‘winning the next moment’. As stated above, there was a genuine acceptance that preparation would be disrupted and that mistakes would be expected. In what was potentially a high-pressured situation, there was a deliberate focus on creating a balance between working hard but also embracing and enjoying the novelty of the situation. A senior player remarked that, “t*here was a good balance of work and enjoyment, the days just flowed better. It was enjoyable to work*”.

As is well-documented, team cohesion is a trait associated with successful teams in sport [46]. Lack of cohesion represented a potential significant challenge with this particular group of players, most of whom had never played together, and many of whom had never played a professional game of rugby. To counterbalance a potential lack of cohesion, the coach’s and senior players’ focus (reflecting the implementation of a dual-management approach) was on creating a task-focused environment by managing the information flow and stripping back the level of detail in the game plan to give (especially the younger) players a freedom to play. One of the coaches mentioned how the game plan had been simplified, “*We gave the players a little bit less detail, were very concise, gave them freedom to play*”. As part of creating a positive, task-focused environment, the coaches’ intention was to manage the level of challenge and the pressure on the players, which less experienced players in particular responded positively to:

“*You had freedom to show what you could do, show your skills, what you had learned up until now, don’t get bogged down on errors and go out and enjoy it. An example of that was a coach saying ‘I just can’t wait to see you play’ gave me great confidence anyway*”.

There was a tangible sense of playing for a cause and playing with a deep sense of purpose among the playing group and the staff. There are numerous examples of successful teams in many different sports that try to connect to a deep sense of purpose or a cause that allows them to elevate their performance by being part of something greater than themselves [47]. It was clear that these steps were built around the identity of Munster, and the coaches deliberately tapped into this fact by positioning the game, “*as a legacy, something special to be a part of, another special Munster moment*”. A senior player who had been involved in many of what would be considered famous games from Munster’s past, felt that it was “*a sense of the old Munster when we were in a corner fighting our way out. It was another chance to add to the Munster history, which it has*”.

As outlined earlier, throughout the entire preparation, there were players and staff that remained in South Africa, others in quarantine at home in Ireland, and the staff and players fortunate enough to be involved in preparing for the game embraced the privilege and responsibility of representing the entire club and its proud history. An academy player captured the sense of responsibility by saying that everyone was, “*representing the players that weren’t there, you were doing a job for them*”.

### 3.3. Impact Post Challenge

In the period following the Munster vs. Wasps game, it was clear that there were both positive and negative impacts on the team. Following the match, there seemed to be an increased vertical alignment along the pathway and an articulation of how interpersonal relationships were significantly enhanced since the game. Some players reported that they were inspired and highly motivated by playing in the game, with others sensing that they were within touching distance of playing in top-level games. Some of the academy and pathway players described how their involvement in the match instilled confidence and belief in their ability to play at senior level. Senior players reported more confidence in the young players coming through the pathway and their ability to make the step into a senior performance environment. Academy staff reported an enhanced level of trust from the senior players and staff, which all contributed to increased vertical alignment between both groups:

“*Positive things that have come from the situation are that we [*academy staff*] are trusted a lot more now by senior staff and by players due to the feedback from the senior players that were here*”.

Both players and staff reported improved interpersonal relationships in the High Performance Centre creating a sense of being one big squad:

“*The dynamic between the younger and senior players is different now, it’s like they are one squad now. The senior players are far more inclusive and engaged with the academy players, any previous divisions are gone*”.

Previous authors [18,48] cited a lack of alignment on the talent development pathway as a barrier to creating an effective, coherent environment. Enhanced trust and improved interpersonal relationships between the academy and senior squad at Munster potentially eliminate, or at least reduce, that barrier and should facilitate greater alignment and coherence on the clubs’ pathway in the future if maintained.

For several academy players, the Munster-Wasps match acted as a catalyst for their development, with many describing how their experience left them inspired, hungry for more and with a renewed confidence in their ability to progress. One academy player reflected that, “*It was my first taste, my first cap, it will probably be my motivation for quite a while*”. While another academy player’s account was that, “*It left you wanting more. It was so much more enjoyable to be centrally involved in the team. I wish it was the same every week*”. An academy coach, in relation to the players that were involved in the game, felt that,

“*Some of them definitely have grown a lot, the confidence that experience has given them, being able play at a level above what they thought they were capable*”.

For those players who helped the team prepare but didn’t make match day 23 there was also a benefit, with the realisation that they were within touching distance,

“*Looking at player A, player B and player C playing, who you train with all the time, you’re thinking that if they are able to do that there’s no reason why I can’t do that as well, it gave you belief, that it’s there for you*”.

In addition, these findings point to the importance of proximal role models on the talent pathway, along with the need to support players prior to and during challenges and transitions to optimise progression [18].

In contrast to the post-event bounce experienced by many players, a small number of players and staff experienced a post-event slump, or anti-climax, after the success of the Wasps game. This is not uncommon with sportspeople who have been involved in what they consider to be major sports events [49]. In the two weeks following the game, the majority of the regular squad members returned from quarantine and were selected in subsequent matchday squads. Many of the young players that had experienced the high of a European Cup game now found themselves with limited opportunities and a return to lower levels of competition, “*There was definitely an anti-climax, I feel like I’m at this level and you’re not playing for the next few weeks, the disappointment because you have a taste of it*”. This feeling wasn’t reserved solely for the players, however, as academy staff also reported the same sense of anti-climax after being centrally involved in preparation for such a big game, “*People were almost disappointed to go back into normal weeks, a come down, after an occasion as special as this. It was different energy*”.

As mentioned earlier, the benefits of the mentoring system employed during the two-week preparation were evident as the season continued, suggesting that there was longevity in the impact of the match, and the match preparations, on systems and behaviours. Many inter-player relationships that have been forged during the build-up to and during the Wasps game have continued to develop since then. One of the senior players talked about, “*texting player A & player B during the U20 six nations, you wouldn’t normally have a relationship with them but now I do and I’ve a genuine interest in how they are getting on*”. Another senior player talked about academy players “*coming up to me looking for advice and if we didn’t go through that experience together that wouldn’t be happening*”. Providing developing players with access to role models in their environment is a factor strongly associated with successful TD environments [2], and the ongoing interpersonal relationships between academy and senior players, arguably a fortuitous spin off from the buddy system employed during game preparation, is a very welcome and positive one.

Finally, senior players noted a renewed confidence in the quality of players that are coming through the Munster system. One senior player stated that we, “*have a different calibre of player coming through now*” and a second senior player addressed the fact that he was previously concerned with the throughput of players from within our pathway, but after this experience he believes the future is bright, “*My biggest thing now is that I’m not worried what’s coming through, I had a big worry but not anymore*”. While the perceived ‘security’ of the club is without doubt of benefit, there are also unseen, and perhaps unexpected, additional advantages to this, in that senior players will be aware of very genuine competition for places in the starting team. It has been well documented that this genuine competition for places amongst a squad is an essential part of a successful TD ecosystem, which can have a direct impact on enhanced levels of performance within teams [50].

### 3.4. General Discussion

The goal of a talent development pathway in sport is to efficiently develop athletes with the ability to perform at the senior level. With this in mind, it is important that talent development environments such as professional sports academies are fit for purpose and promote talent through the pipeline. This study took advantage of a naturally occurring (but highly atypical) challenge (i.e., Munster Rugby’s loss of 48 players and staff due to COVID-19 restrictions for a vital European Rugby Cup fixture) to pressure test the efficacy and effectiveness of a professional rugby club’s talent pathway.

This scenario provided a real-life case study from which to examine the groundwork, principles, and practices that underpinned the talent development environment and how navigating a significant challenge provided opportunities for growth and development of both players and staff following the experience [51] Findings from this study clearly demonstrate the importance of an effective talent pathway in supporting performance objectives at senior level [9] and the need for coherent and aligned systems on the pathway that support players to be ready for the next step in their journey. Arguably, this may be even more important in (relatively) less resourced sports such as rugby union clubs, where squad size and depth is constrained by budget limitations [1].

At a system’s level, the results point to the importance of vertical and horizontal coherence in the TDE, with development and performance clearly overlapping on the talent pathway [5]. In these particular circumstances, the academy (development) players performed and the senior (performance) players developed. This raises an interesting question in terms of ‘where does performance begin and development end?’. Given the dynamic environment of elite sport, and the need for high potential young players to optimise development opportunities when they arise, it would seem that the performance-development dichotomy is more an academic delineation rather than an applied reality. Given the performance pressures and competitive characteristics that are inevitable features of high performance, the need for a TD pathway to be both efficient (getting players there as quickly as possible) and effective (getting players there as ‘ready’ as possible), points to the importance of a TD pathway having both short-term and longterm objectives [12].

Preparing for and learning from challenges is central to the talent systems approach [23]. The role of challenge as a development catalyst is well established [52], provided the TD system supports its players to proactively prepare for challenge and, just as importantly, players are managed post challenge to ensure learning and growth are facilitated moving forward [51]. There exists an abundance of literature examining the role of challenge in TD from an individual psychological perspective [22], but the findings of this current study highlight the importance of both the playe*r* being prepared and the TD system being prepared to proactively support its athletes. Our findings support that the steps taken to connect the pathway by (a) aligning the key stakeholders from top to bottom, and (b) increasing coherence between different groups at various stages, were influential in navigating the significant challenges that were encountered. The importance of empowering players [39], creating a high-performing, positive, task-focused environment [37], utilising role models [53], and establishing trust, connections, and a deep purpose in a playing group, were all considered influential in achieving a successful outcome in the Wasps-Munster match at the centre of this study.

Given the considerable discussion about the role of TD in sport, from a system perspective, examining the effectiveness and efficiency of the pathway holds both academic and applied interest moving forward. Effective TDEs are characterised as deploying long-term aims and methods and being structured against long-term agendas [4]. In his study involving the Ajax soccer academy, Larsen [54], highlighted several environmental success factors, including the focus on long-term development rather than winning the next match. Ajax provided opportunities for individual long-term development in preference to short-term success. To reinforce this philosophy, the academy complex is very aptly named ‘De Toekomst’, which translates to ‘The Future’.

In this current study, the findings point to an obvious overlap in the pathway between development and performance phases. This creates potential conflict between a ‘win later’ development approach and a ‘win now’ performance philosophy, usually associated with a much shorter-term agenda. Often, development coaching is not clearly conceptualised, and again, as explored above, it can be difficult to identify where development coaching ends and performance coaching begins. There is a lack of research into the nuances of development coaching versus performance coaching, and more research is needed to investigate the in situ contextual practice of coaches working in successful TDE’s. Such research would serve to inform and develop coaching practice *for* the coach, rather than the coach, in the TD space. With increasing pressure in professional sport to get more players ready to perform at the top level, even earlier in their development, continuing to explore how we optimise the design and operation of effective TD pathways is critical, along with further research investigating, informing, and developing practices of (development) coaches.

Finally, although the insider perspective provided by the first author’s position added richness to the data, it must be acknowledged that there are some limitations to this approach. Such a scenario can lead to cultural bias, and due to the first author’s position as academy manager, there may also be a degree of ‘impression management’ among the participants during the interviews. In addition, this was one event, in one season, on one specific pathway, so it is important that practitioners question why this practice works in this context with this particular group, rather than assuming to generalise. As such, caution needs to be exercised when interpreting findings and applying them to other contexts and environments.

## Figures and Tables

**Table 1:** Thematic Analysis.

Higher Order Themes	Lower Order Themes	Raw Data Themes
Groundwork to establish alignment and coherence on the pathway	Integration/alignment of academy and senior team (players and staff)	Training programme alignment
		Senior and academy coaches alignment
		Social integration between academy and first team
		Connections and communications with senior coaches
	Connecting the pathway	Coaching up and down the pathway
		Shared mental models/common coaching framework
		Relationships between academy coaches and pathway players
	Creating a high performing environment	High challenge support environment
		Clear purpose and vision guiding behaviours/practices
	Horizontal alignment with key stakeholders in the TDE	Links to clubs and mutual support
		Practices to inform player selection
		Increased visibility of Munster coaches at club games
Preparation for the Munster-Wasps game	Dual management— Player empowerment and responsibility	Empowering senior players
		Building trust between coaches and players
		Shared ownership of game preparation
		Player led sessions and meetings
	Squad integration, building trust and connections	Getting on the same page quickly
		Connecting the squad
	Creating a positive task focused environment	Creating balance between work and enjoyment
		Managing information flow
		Managing pressure and challenge
		Excitement to embrace the challenge
	Role models and mentoring	Buddy system
		Inspired by role models
		Support from senior players
	Playing with a cause and a deep sense of purpose	Creating a legacy, a Munster moment
		Clear sense of identity and history
		Representing the whole club
Impact post challenge	Inspired and highly motivated players	Motivation from first cap
		Inspired and hungry for more
	Increased vertical alignment and interpersonal relationships between staff and players	Acceptance as a Munster senior player
		Established trust between senior and academy staff
		Increased engagement and inclusivity in the HPC
		Stronger relationships across the club
		Connection through shared experiences
	Newfound confidence and belief to play at this level	Confidence in own ability
		Belief that opportunity is within touching distance
	Post event anti-climax	Disappointment with limited playing opportunities post Wasps game
		Academy staff experienced an anti-climax slump
	Confidence that the future is bright	Confidence in players coming through pathway
		Belief in young players ability

## Data Availability

Not applicable, as the study includes data protected by the Norwegian Personal Data Act and The General Data Protection Regulation from the EU (GDPR).

## References

[1] Till K., Baker J. (2020). Challenges and [Possible] Solutions to Optimizing Talent Identification and Development in Sport. Front. Psychol..

[2] Henriksen K., Stambulova N., Roessler K.K. (2010). Holistic approach to athletic talent development environments: A successful sailing milieu. Psychol. Sport Exerc..

[3] Gledhill A., Harwood C., Forsdyke D. (2017). Psychosocial factors associated with talent development in football: A systematic review. Psychol. Sport Exerc..

[4] Martindale R.J., Collins D., Daubney J. (2005). Talent Development: A Guide for Practice and Research Within Sport. Quest.

[5] Taylor J., Collins D. (2021). Getting in the Way: Investigating Barriers to Optimizing Talent Development Experience. J. Expertise.

[6] Williams G.G., MacNamara Á. (2020). Coaching on the Talent Pathway: Understanding the Influence of Developmental Experiences on Coaching Philosophy. Int. Sport Coach. J..

[7] Giacobbi P.R., Poczwardowski A., Hager P.F. (2005). A Pragmatic Research Philosophy for Applied Sport Psychology. Sport Psychol..

[8] Collins D., Kamin S., Murphy S.M. (2012). The performance coach. The Oxford Handbook of Sport and Performance Psychology.

[9] Webb V., Collins D., Cruickshank A. (2016). Aligning the talent pathway: Exploring the role and mechanisms of coherence in development. J. Sports Sci..

[10] Johnson D.K.N., Ali A. (2004). A Tale of Two Seasons: Participation and Medal Counts at the Summer and Winter Olympic Games. Soc. Sci. Q..

[11] Bjørndal C.T., Ronglan L.T. (2018). Orchestrating talent development: Youth players’ developmental experiences in Scandinavian team sports. Sports Coach. Rev..

[12] Martindale R.J.J., Collins D., Abraham A. (2007). Effective Talent Development: The Elite Coach Perspective in UK Sport. J. Appl. Sport Psychol..

[13] Mills A., Butt J., Maynard I., Harwood C. (2014). Examining the Development Environments of Elite English Football Academies: The Players’ Perspective. Int. J. Sports Sci. Coach..

[14] Larsen C.H., Alfermann D., Henriksen K., Christensen M.K. (2013). Successful talent development in soccer: The characteristics of the environment. Sport Exerc. Perform. Psychol..

[15] McComb S., Simpson V. (2014). The concept of shared mental models in healthcare collaboration. J. Adv. Nurs..

[16] Ivarsson A., Stenling A., Fallby J., Johnson U., Borg E., Johansson G. (2015). The predictive ability of the talent development environment on youth elite football players’ well-being: A person-centered approach. Psychol. Sport Exerc..

[17] Curran O., MacNamara Á., Passmore D. (2021). Singing off the same hymn sheet? Examining coherence in a talent development pathway (part 1). J. Sports Sci..

[18] Collins D., MacNamara Á. (2012). The Rocky Road to the Top. Sports Med..

[19] Wylleman P., Reints A. (2010). A Lifetime perspective on the career of talented an elite athletes: Perspectives of high-intensity sports. Scand. J. Sci. Med. Sports.

[20] Wylleman P., Lavallee D. (2004). A developmental perspective on transitions faced by athletes. Developmental Sport and Exercise Psychology: A Lifespan Perspective.

[21] Stambulova N.B. (2000). Athlete’s crises: A developmental perspective. Int. J. Sport Psychol..

[22] MacNamara A., Button A., Collins D. (2010). The Role of Psychological Characteristics in Facilitating the Pathway to Elite Performance Part 1: Identifying Mental Skills and Behaviors. Sport Psychol..

[23] Savage J., Collins D., Cruickshank A. (2017). Exploring Traumas in the Development of Talent: What Are They, What Do They Do, and What Do They Require?. J. Appl. Sport Psychol..

[24] Hafiz K., Baxter P., Jack S. (2008). Case study example Qualitative Case Study Methodology: Study Design and Implementation for Novice Researchers. The Qualitative Report.

[25] Gesbert V., von Roten F.C., Hauw D. (2021). Reviewing the role of the environment in the talent development of a professional soccer club. PLoS ONE.

[26] Nash C., Taylor J. (2021). ‘Just Let Them Play’: Complex Dynamics in Youth Sport, Why It Isn’t So Simple. Front. Psychol..

[27] Smith B., McGannon K. (2018). Developing rigor in qualitative research: Problems and opportunities within sport and exercise psychology. Int. Rev. Sport Exerc. Psychol..

[28] Gibbs G. (2007). Analyzing Qualitative Data.

[29] Fetterman D.M. (2010). Ethnography: Step by Step.

[30] Glaser B., Srauss A. (1967). The Discovery of Grounded Theory.

[31] Lincoln Y.S., Guba E.G. (1985). Naturalistic Inquiry.

[32] Mason J. (1996). Qualitative Researching.

[33] Bradshaw C., Atkinson S., Doody O. (2017). Employing a Qualitative Description Approach in Health Care Research. Glob. Qual. Nurs. Res..

[34] Greene M. (2014). On the Inside Looking In: Methodological Insights and Challenges in Conducting Qualitative Insider Research. Qual. Rep..

[35] Berger R. (2015). Now I see it, now I don’t: Researcher’s position and reflexivity in qualitative research. Qual. Res..

[36] Mills A., Butt J., Maynard I., Harwood C. (2012). Identifying factors perceived to influence the development of elite youth football academy players. J. Sports Sci..

[37] Lara-Bercial S., Mallett C.J. (2016). The Practices and Developmental Pathways of Professional and Olympic Serial Winning Coaches. Int. Sport Coach. J..

[38] Hall A.J., Jones L., Martindale R.J. (2019). The Talent Development Environment Questionnaire as a Tool to Drive Excellence in Elite Sport Environments. Int. Sport Coach. J..

[39] Hodge K., Henry G., Smith W. (2014). A Case Study of Excellence in Elite Sport: Motivational Climate in a World Champion Team. Sport Psychol..

[40] Lyons M., Rynne S.B., Mallett C.J. (2012). Reflective Practice International and Multidisciplinary Perspectives. Reflection and the art of coaching: Fostering high-performance in Olympic Ski Cross. Reflective Pract..

[41] Chan J.T., Mallett C.J. (2011). The Value of Emotional Intelligence for High Performance Coaching. Int. J. Sports Sci. Coach..

[42] Bushmaker T., Lund M., Meyer J., Mcnulty E., Wait K., Braun S. (2019). Effectiveness of a Buddy System on Strength Training, Adherence, Confidence, and Perceived Health in College Females. Int. J. Res. Ex. Phys..

[43] Henriksen K., Stambulova N., Roessler K.K. (2010). Successful talent development in track and field: Considering the role of environment. Scand. J. Med. Sci. Sports.

[44] Adronikos G., Westbury T., Brazo-Sayavera J., Olivares P., Martindale R. (2021). Factors contributing to the quality of the junior-senior transition in Greek athletes. Int. J. Sport Exerc. Psychol..

[45] Jackson S.A., Roberts G.C. (1992). Positive Perfonnance States of Athletes: Toward a Conceptual Understanding of Peak Performance. Sporl Psychol..

[46] Bruner M.W., Eys M.A., Wilson K.S., Côté J. (2014). Group cohesion and positive youth development in team sport athletes. Sport Exerc. Perform. Psychol..

[47] Johnson T., Martin A.J., Palmer F.R., Watson G., Ramsey P. (2013). A Core Value of Pride in Winning: The All Blacks’ Team Culture and Legacy. Int. J. Sport Soc..

[48] Taylor J., Collins D. (2020). The Highs and the Lows—Exploring the Nature of Optimally Impactful Development Experiences on the Talent Pathway. Sport Psychol..

[49] Bradshaw H., Howells K., Lucassen M. (2021). Abandoned to manage the post-Olympic blues: Olympians reflect on their experiences and the need for a change. Qual. Res. Sport Exerc. Health.

[50] Passos P., Araújo D., Davids K. (2016). Competitiveness and the Process of Co-adaptation in Team Sport Performance. Front. Psychol..

[51] Taylor J., Collins D. (2021). Navigating the winds of change on the smooth sea—The interaction of feedback and emotional disruption on the talent pathway. J. Appl. Sport Psychol..

[52] Taylor J., Collins D. (2019). Shoulda, Coulda, Didnae—Why Don’t High-Potential Players Make it?. Sport Psychol..

[53] Henriksen K., Larsen C.H., Christensen M.K. (2014). Looking at success from its opposite pole: The case of a talent development golf environment in Denmark. Int. J. Sport Exerc. Psychol..

[54] Larsen C.H., Storm L.K., Sæther S.A., Pyrdol N., Henriksen K. (2020). A world class academy in professional football: The Case of Ajax Amsterdam. Scand. J. Sport Exerc. Psychol..

